# Impact of peer-led quality improvement networks on quality of inpatient mental health care: study protocol for a cluster randomized controlled trial

**DOI:** 10.1186/s12888-016-1040-1

**Published:** 2016-09-21

**Authors:** Lina Aimola, Sarah Jasim, Neeraj Tripathi, Sarah Tucker, Adrian Worrall, Alan Quirk, Mike J. Crawford

**Affiliations:** 1 College Centre for Quality Improvement, The Royal College of Psychiatrists, 21 Prescot St, London, E1 8BB London, UK; 2 Centre for Mental Health, Hammersmith Campus, Imperial College London, 7th Floor Commonwealth Building, Du Cane Road, W12 0NN London, UK; 3 Southview Low Secure Unit, Sussex Partnership NHS Foundation Trust, The Drive, Hellingly, Hailsham, BN27 4ER East Sussex UK; 4 Association of Group and Individual Psychotherapy, 1 Fairbridge Road, N19 3EW London, UK

**Keywords:** Quality networks, Peer-review, Randomized controlled trial, Forensic mental health

## Abstract

**Background:**

Quality improvement networks are peer-led programmes in which members of the network assess the quality of care colleagues provide according to agreed standards of practice. These networks aim to help members identify areas of service provision that could be improved and share good practice. Despite the widespread use of peer-led quality improvement networks, there is very little information about their impact. We are conducting a cluster randomized controlled trial of a quality improvement network for low-secure mental health wards to examine the impact of membership on the process and outcomes of care over a 12 month period.

**Methods:**

Standalone low secure units in England and Wales that expressed an interest in joining the quality improvement network were recruited for the study from 2012 to 2014. Thirty-eight units were randomly allocated to either the active intervention (participation in the network *n* = 18) or a control arm (delayed participation in the network *n* = 20). Using a 5 % significance level and 90 % power, it was calculated that a sample size of 60 wards was required taking into account a 10 % drop out. A total of 75 wards were assessed at baseline and 8 wards dropped out the study before the data collection at follow up. Researchers masked to the allocation status of the units assessed all study outcomes at baseline and follow-up 12 months later. The primary outcome is the quality of the physical environment and facilities on the wards. The secondary outcomes are: safety of the ward, patient-rated satisfaction with care and mental well-being, staff burnout, training and supervision. Relative to control wards, it is hypothesized that the quality of the physical environment and facilities will be higher on wards in the active arm of the trial 12 months after randomization.

**Discussion:**

To our knowledge, this is the first randomized evaluation of a peer-led quality improvement network that has examined the impact of participation on both patient-level and service-level outcomes. The study has the potential to help shape future efforts to improve the quality of inpatient care.

**Trial registration:**

Current Controlled Trials ISRCTN79614916. Retrospectively registered 28 March 2014]

## Background

Quality networks aim to enhance standards of health care by engaging and supporting clinicians and managers to assess and improve the quality of the care they provide [1]. By assessing quality of care against recommended standards and providing feedback on service quality to front-line staff, quality networks have much in common with accreditation schemes. However, in quality networks services do not work towards achieving accreditation for the care they provide. Rather, they receive suggestions and support for initiating changes to services and to keep front-line staff engaged in efforts to improve service quality. Over recent years the number of quality networks and accreditation programmes has substantially increased [2]. Despite this, there is little evidence to judge how effective they are, and the impact they have on patient outcomes is largely unknown.

Data from observational studies of services that participate in quality networks show that they achieve higher standards of care over time [3–5]. However, comparative data from services that do not take part in such networks have rarely been gathered. A greater amount of research has been conducted into the impact of accreditation programmes. A systematic review of the impact of accreditation programs found some evidence from observational studies that such programmes improve staff perceptions of service quality [6]. Consistent findings about impact on patient outcomes were not found.

To date, two randomized controlled trials have been carried out to explore whether peer-led quality improvement programs enhance the quality of patient care [7, 8]. The first study was conducted in acute hospitals in KwaZulu-Natal, South Africa. Over the course of two years, greater improvements in standards of care were seen in hospitals that were randomized to participation in an accreditation programme. However, differences in clinical records keeping, hospital sanitation and patient satisfaction with care were not found. While this study demonstrated that experimental studies of peer-led quality improvement initiatives are feasible, its limitations, including a small sample size and inconsistent implementation of the accreditation programme limits generalisability to other clinical settings. In addition, hospitals that were randomized to control treatment appear to have had limited exposure to other quality improvement initiatives. In Europe, North America and other industrialised economies health services are usually involved in a range of other quality improvement initiatives, such as local audits or statutory inspection. In this context, Roberts and colleagues [8] conducted a randomized trial to explore the added value of participation in a peer-led quality improvement initiative in the United Kingdom. In this study, hospitals admitting patients with acute Chronic Obstructive Pulmonary Disease (COPD) were randomized to the intervention arm of the trial that received a reciprocal peer-review and a control arm that continued to undertake COPD service development within normal processes. A number of quality indicators drawn from national guidelines were used to measure service outcomes as well as four key areas of service provision. Participating services had to complete a self-assessment baseline pro-forma describing the service provision and attainment (met in full, partially met, not met at all) of the quality indicators. This document was used to direct discussions and record observations during the peer-review visit. After each peer-review visit, the intervention units received a final report and agreed action plans for service development. All services were asked to complete a change diary 12 months after all the peer-review visits were completed to provide information about major service changes occurring during the year that could be related to their involvement in the study or not. Follow-up data at 12 months on those aspects of care assessed at baseline were collected as part of the UK National COPD Audit. The results showed that the compliance of the services in the intervention group with the quality indicators assessed was only marginally higher than that shown by the control group. In contrast, qualitative data suggested many benefits of the peer-review in most intervention units and some control teams [8]. One limitation of this study was that the data collected at baseline relied entirely on the services’ self-assessment. In addition, the team did not assess whether the peer review process had any impact on patients’ experience of their care.

The Royal College of Psychiatrists’ Centre for Quality Improvement runs a range of peer-led quality improvement programmes (see: http://www.rcpsych.ac.uk/researchandtrainingunit/centreforqualityimprovement.aspx). The setting up of a new quality improvement network starts with the development of service standards that are based on recommendations made by professional bodies and organisations such as the National Institute for Health and Clinical Excellence [9, 10]. Front-line clinicians and service users and carers are all involved in the development of these standards. Services that choose to participate in a network are sent materials necessary to conduct a self-review (where they indicate whether they believe that they meet or do not meet agreed standards of care). The self-review is followed by an independent assessment led by a multi-professional team of trained peers who work in similar clinical settings. Service users and carers also take part in these assessments [1]. Discrepancies between the self-review and the peer review data are discussed and feedback on service quality is then provided to each service after each visit. Clinical teams are sent a final report summarising the services’ achievements and areas of improvement and are supported to develop an action plan aimed at making improvements to service quality. Participation in a quality network also offers access to online discussion groups, newsletters, workshops and an annual forum. These activities encourage services to share good practice and find solutions to the challenges they share.

Essentially, network membership entails committing to a culture of openness, sharing and enquiry through a supportive peer-led process with the common goal for all members to improve quality of care. All members are required to engage actively in all stages of the peer-review cycle (self-review, peer-review, attendance of annual forum) and expected to use the results of reviews to develop action plans to achieve year on year improvement. Members are also expected to share their results throughout their services as well as with key stakeholders, including health and local authorities, those making referrals to their services and local patient and carer groups.

Secure inpatient forensic mental health units are specialist services for people with mental health problems who have either committed a criminal offence or whose challenging behaviour requires a level of security that is higher than that provided in mainstream adult mental health services. They are called ‘secure’ because the freedom of people treated on these units is restricted by mental health legislation. Inpatient forensic mental health services aim to treat people’s mental health problems and ensure the safety of patients and the public by monitoring risk, preventing absconsions and providing support and supervision while on the unit and on agreed periods of leave. When the decision was made to set up a new peer-review network for low secure inpatient forensic units, we built in a randomized evaluation in which low secure units were randomly allocated to immediate or delayed participation in the quality network 12 months later. The low-secure network aims to help individual services identify areas where their practice falls below national standards and to share good practice aimed at improving the quality of care they provide. The standards used in the network cover key aspects of physical and relational security, the interventions and treatments patients are offered, the quality of the physical environment, training, supervision and support for staff the governance of services including the way that adverse incidents are reported and managed. We hypothesised that through helping teams focus on these areas of care, membership of the network would help them identity where these elements of care could be improved and that this in turn would impact on the health, well-being and experience of the patients treated on these units.

### Aim of the study

The aim of the study is to assess the impact of membership of the quality improvement network for Low Secure Units on the quality of care that people receive.

### Hypotheses

- The primary hypothesis is that the quality of the physical environment and facilities in wards that participate in the network will be higher 12 months after randomization than in those wards that do not participate.
- The secondary hypotheses are that, 12 months after randomization, wards that participate in the network will have:

i) Higher levels of safety
ii) Higher levels of patient satisfaction
iii) Higher levels of patient mental well-being
iv) Lower levels of staff burnout
v) Higher levels of training and supervision for staff

## Methods

### Trial design

The study is a two-armed, parallel group, researcher-masked, randomized controlled trial of early or late participation in a quality network. Units randomized to late participation in the network had the opportunity to join the network once all follow-up data were collected 12 months after randomization.

### Study setting and sample

**Fig. 1:**
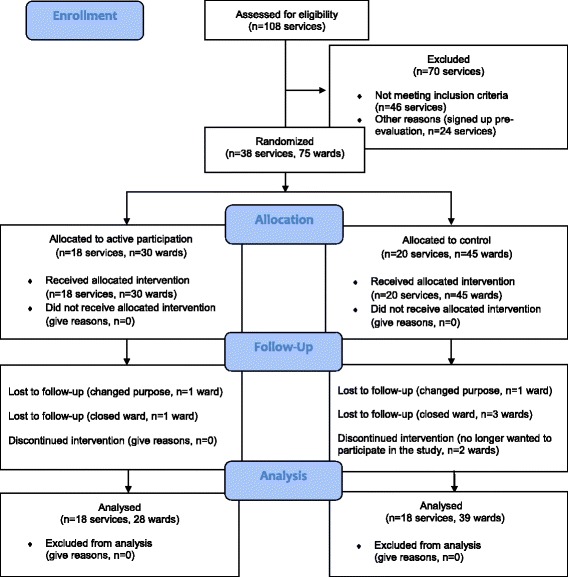
CONSORT 2010 Flow Diagram

Between June 2012 and June 2014, 108 low secure units were recruited to join the low secure quality network. Seventy of these units were ineligible for the study because either a) were located on the same premises as Medium Secure Forensic units that already participate in a quality network for these services (*n* = 46) or b) joined the network before the study was set up (*n* = 24). The reason to exclude the combined sites was to avoid that some staff employed across both sets of units may already have implemented changes based on their experience of the medium secure network. From the initial sample, 38 standalone units were eligible for the study and agreed to take part in the randomised trial. To minimise cross-level contamination between control and treatment group, only the members of the network could have access and share resources on the quality network website within a password protected area. The networks’ newsletters and the annual reports were made available online only after all the data collection for the whole study was completed (see Fig. 1)

.

### Study interventions

#### The quality improvement network for low secure inpatient mental health units

Standards for the network were developed by the Quality Network for Forensic Mental Health Services team (QNFMHS) directly from the ‘Low Secure Services: Good practice commissioning guide consultation draft Department of Health [11]. Some standards from the ‘Implementation Criteria for Recommended Specification: Adult Medium Secure Units’ [9] were also included. To finalise the development of the standards, the Quality Network team consulted an expert standards steering committee comprising commissioners, providers and ex-users of forensic mental health services regarding clarity, measurability and importance. In addition, a final and wider standard consultation conference was held where experts in forensic mental health (including service user representatives and carers) provided further feedback on the standards. This feedback was then incorporated in the final version of the document [12]. Forming the foundation of the iterative annual review cycle, the standards provide an accessible way for services to actively engage in on-going service development towards implementing the Department of Health recommendations. These standards form the basis of the self-review that all wards in the active arm of the trial were asked to undertake at the start of the review cycle. Having completed a self-review, arrangements were made for all services to be visited for a peer-review. Peer-reviewers were recruited from staff working in the service members, and all lead reviewers were provided with a lead reviewer training prior to their first visit. This training was delivered by the Forensic Quality Network team. At the peer-review visit, each service is required to i) arrange for the review team to be escorted for a tour of the service to assess the patients’ facilities ii) organise service users interviews, iii) provide the documentation for a service policy check in relation to the aspects of care covered in the standards, iv) organise 3 separate staff meetings with senior managers, the multidisciplinary team (MDT) and front line staff. At the end of the visit, the review team provides feedback to managers and staff representatives to highlight discrepancies between the self-assessment and the peer-review data, the service’s achievements as well as areas that need improvements. After the peer-review stage, each service is provided with a local report that compiles self and peer-review data and shows the extent to which the service meets the standards.

When all member services have completed the peer-review phase, an annual national report of the aggregated findings is written to enable benchmarking with other services and reflect on their practice. The final stage of the programme involves action planning, an invitation to an annual forum and access to newsletters and an e-mail discussion group. At the annual forum members of the network have an opportunity to hear about challenges faced by similar units and share examples of how different units have tried to meet these challenges. All the material produced by the network (e.g., annual reports, newsletters) is made available to members via a password-protected webpage.

#### Control treatment

Aside from participating in the network 12 months after randomization, all wards in the control arm of the trial were free to carry out any other quality improvement initiatives as per normal. Data about all quality improvement initiatives that wards used in the 12 month follow up period were collected. These may include local reviews, audits and the possibility of inspection by statutory bodies. Twelve months after randomization, all control wards were invited to join the quality network once all follow-up data had been collected.

#### Fidelity to active treatment

During the review cycle, the managers who co-ordinated the forensic quality network monitored the involvement in the network of all units in the active arm of the trial. This involvement included: i) two or more members of staff attending reviewer training, ii) completion of the self-review workbook at least six weeks before the date of the peer-review visit, iii) completion of a peer-review visit according to timetable, iv) participation in discussion groups and v) attendance by one or more members of staff at the annual forum.

### Measures

#### Primary outcome measure

The primary outcome was the quality of the physical environment and facilities on the wards which was assessed using a validated ‘environmental checklist’ named the QELS (Quality of Environment in Low secure Services). The QELS checklist assesses 10 domains of quality of care and generates a score between zero and 100, with higher scores indicating a higher quality of service provision relative to the physical environment (see text Table 1) [11]. Tests of the psychometric properties of the environmental checklist have shown that it has good inter-rater reliability and concurrent validity. The checklist was completed by a member of the research team who visited each ward to collect the study data. During these visits the researcher was always masked to allocation status of the unit (see randomization and blinding section for details).

**Table 1:** Items of the QELS checklist

1. Whether the service has an external perimeter that meets the standards for security.	
2. Whether there are separate, accessible and appropriately furnished facilities for visitors.	
3. Whether all visitors, staff and patients access the ward via airlock.	
4. Whether there are any ligature points on the ward.	
5. Whether the ward has a multi-faith room accessible and appropriate for use by all patients.	
6. Whether the ward has an up to standards seclusion room.	
7. Whether the ward has a de-escalation room.	
8. Whether patients’ bedrooms are designed to maintain safety.	
9. Whether there is a variety of recreational facilities accessible to patients.	
10. Whether there is a variety of occupational facilities accessible to patients.	

#### Secondary outcome measures

i) Safety of the wards was assessed by the visiting researcher who interviewed the manager of each ward and collecting data on: number of absconsions and untoward incidents during the previous 6 months [13], proportion of patients and staff who reported being assaulted in the previous 3 months and patient and staff experience of feeling safe on the ward.
ii) Service user satisfaction with care was assessed using a modified version of the Patient Satisfaction Questionnaire (PSQ) [14]. The questionnaire was modified with the approval of the team that designed the measure by changing one item to make it suitable for assessing patient experience of satisfaction with inpatient treatment (the item on “satisfaction with the place and time of the appointments” was changed to” satisfaction with the place you live” (e.g., bedroom, common areas). This questionnaire was embedded in a patient survey used in the study which included also questions on the patients’ access to community services and involvement in planning the care they receive. The patient survey was administrated by the visiting researcher on site with the help of staff members when needed to facilitate engagement with the patients.
iii) Mental wellbeing of patients on wards was assessed using the Short Warwick-Edinburgh Mental Well-being Scale (SWEMWBS) [15] which was also embedded in the patient survey. The SWEMWBS was used in this study because of its brevity and robust psychometric properties [15].
iv) Staff burnout was measured using the Maslach Burnout Inventory (MBI) [16, 17]. The MBI is a leading, validated measures of burnout used extensively in human services, education and business. This survey consists of 22 items that create three general subscales assessing: emotional exhaustion, depersonalization and personal accomplishment. Numerous psychometric analyses showed that the scale has both high reliability and validity as a measure of burnout [18]. The MBI was included in a staff survey used in the study which was distributed to staff to complete by the visiting researcher.
v) Staff members on the ward were asked to indicate the extent to which the training and the supervision they receive was adequate to carry out their job. These questions were included in the staff survey distributed by the researcher.

### Study procedures

#### Recruitment and consent procedures

The recruitment of the low secure units was carried out by the Quality Network for Forensic Mental Health Services team who publicised the study on the network’s website. Recruitment took place over three years as part of an annual cycle of recruitment, self-review and peer review between June 2012 and June 2014. At the beginning of each peer-review cycle, the eligible units that agreed to take part in the network were randomized to the active or control arm of the trial (see study setting and sample section for details on eligibility). Thirty-eight units in total took part in the study (75 wards). Each unit nominated a link person to communicate with the research team to arrange the data collection visit. The link person was in charge of circulating the study information sheet to staff and patients at least two weeks prior the data collection visit by the researcher and coordinate the logistic of the visit between the researcher, staff and patients on the wards. The researcher was escorted by staff members on the day of the visit to observe the wards’ facilities and complete the environmental checklist. During the escorted visit the researcher engaged with staff and patients by providing further details about the study and sought informed consent to complete the study survey from those who were willing to participate. The patients who agreed to participate were assisted by the researcher or staff to fill in their survey if needed. In order to maximize completeness of data collection, the research team made every effort to monitor participants’ retention from baseline to follow-up. Retention strategies included consistent and frequent contact of the research team with service in preparation for the data collection visits.

#### Randomization and blinding

Some wards were part of a unit that included other wards on the same site. While there may be differences in services provided by different wards on the same unit, management is often shared and we judged that there was considerable potential for contamination of the effects of the intervention across wards on the same unit. We therefore randomized units rather than wards. The Quality Network team emailed the contact details of eligible services to an independent team based at Imperial College London which was in charge of the randomization process. This team used a web-based randomization programme and a randomization ratio of 1:1, early versus delayed participation in the network. Randomization was stratified according to the size of the unit (whether they contained up to four, or more than four wards). Following randomization of the 38 eligible units, 18 (47 %) were allocated by the independent team to the active arm and 20 (53 %) to the control arm of the study. The list of early and late participation units was then sent back to the Quality Network team that informed the units of their membership status.

Once all the units were informed of their allocation status, the quality network team sent the research team the full list of units enrolled for the study without any reference to their group membership. The research group then made contact with the units and arranged for baseline data collection. The units’ link person and all staff members were repeatedly reminded that the researcher collecting study data must not be told which arm of the trial they were in. In the event of accidental unmasking of a researcher, all further data were collected by a second researcher who was masked to the allocation status of the unit.

#### Collection of study data

Baseline data were gathered by the researchers masked to allocation status of the units via direct observation of each individual ward and its facilities, an interview with the ward manager (to collate routine data on incidents and wards costs), and surveys of staff and service users. The unit’s link person produced a timetable for the data collection visit that specified what time and where each component of the visit would take place (e.g., managers’ interview, tour of the wards, meetings with patients and staff). All service users were asked to take part in the survey except those where clinical staff judged that their current mental state meant they should not be approached by a member of the research team. Participants were given the option of completing and returning the questionnaires on the day of the assessment or sending them back to the research team in a pre-paid envelope. Pre-paid envelopes and blank questionnaires to complete were left for those staff members that were not scheduled on shift during the visit and for service users on community leave. No identifiable information about staff and service users were recorded. Twelve months after the collection of baseline data, a member of the research team who was again masked to allocation status of the unit arranged to visit each site again for a follow-up assessment. The instruments used at baseline were administrated for a second time at follow-up in the same manner.

#### Sample size

The sample size for the study was based on the primary hypothesis: the quality of the physical environment and facilities in wards that participate in the network would be higher 12 months after randomization than in those wards that do not participate in the network. A previous investigation with medium secure wards that took part in the quality network showed an increase in the proportion of standards met equivalent to a 10 % increase during the first year of membership of the network [19].

Assuming no change in outcome in the control arm, the sample size was based on detecting a 10 % difference between groups, equivalent to 10 units of the environmental checklist score. The sample size was calculated assuming an ANCOVA analysis, with the baseline scores used as the covariate. Based on preliminary data, a correlation of 0.53 between baseline and outcome scores was assumed. A standard deviation of 13.3 for the primary outcome was assumed (estimated using baseline data from the first 32 wards that took part in the trial). Using a 5 % significance level and 90 % power, it was calculated that 54 wards were required (27 in the active arm and 27 in the control arm of the trial). To take account of 10 % drop out from the study, we increased the sample size to 60 wards.

#### Data management and analysis

All data were entered electronically into a database. Once data collection was completed, 100 % of the scores of the primary outcome measure were double checked against source data for accuracy. All analysis will follow the intention-to-treat principle. Descriptive analyses including tables and graphs of baseline organisational processes and clinical outcomes will be produced for each of the two arms of the trial.

The scores of the primary outcome measure (QELS checklist scores at 12 months) will be compared between the two groups using analysis of covariance (ANCOVA). The scores at 12 month will be considered as the outcome measure, with the wards’ scores at baseline used as a covariate.

The scores of the secondary outcome variables measured at the ward level and on continuous scales (e.g., number of untoward incidents), will be analysed using the unpaired *t*-test or Mann–Whitney test depending on the data distribution. Most secondary outcome variables are measured at the staff or patient level (e.g., staff burnout, service users mental wellbeing and satisfaction with care). It is anticipated that there will be clustering of outcomes as a result of participants receiving interventions on different wards. Such clustering violates the assumption that observed outcomes of participants are independent and can result in increased standard errors [20, 21]. To take account of this, these outcomes will be analysed using a two level multilevel regression models with patients/staff at level one and the wards as the second level. When measured, baseline values of each outcome will be used as a covariate in the analysis. Additional exploratory analysis will investigate possible predictors of the primary outcome (environmental checklist scores at 12 months). This analysis will be performed using multiple linear regression.

## Discussion

This study provides an opportunity to explore the impact of participation in a peer network on the quality of inpatient mental health services. By comparing the quality of the physical environment and facilities of units that do and do not take part in a network we will able to find out what, if any, benefit membership of a peer network delivers. In addition, the study will explore whether participation in a quality network has any impact on staff and patient outcomes. To our knowledge this is the first time that a randomized evaluation of participation in a peer-led network has been conducted that will examine patient and staff safety, patient experience and the clinical effectiveness of care that patients receive.

Quality networks are one of a broad range of initiatives that services may use to try to improve the quality of care they provide. All such initiatives have costs as well as potential benefits, both in terms of direct costs of delivering the programme and time spent by staff completing self and peer reviews. Strengths of this study are that it is adequately powered to examine clinically important differences in service quality and the wide geographical area that the units were recruited from. By integrating the trial into the development of a new quality improvement network we ensured that nearly all eligible services took part in the study.

Our primary outcome is designed to assess the quality of the physical environment and facilities on participating wards. This aspect of services is one which a previous observational study has suggested is amenable to change within the first year of participation in a quality improvement network [19]. Limitations inherent in the study design are the relatively short follow-up period and changes in patients and staff at wards that may limit our ability to assess the impact of participation in the network on patient and staff outcomes. Staff involved in setting up the network were concerned about withholding membership from control wards. Wards felt that a delay of more than a year would be unacceptable. This means that we will only be able to examine the impact of the first year of membership of the network and we will not be able to examine any benefits associated with longer-term participation. Given turnover in staff and patients at study wards, we are not able to compare changes in staff burnout, or patient mental health and satisfaction with treatment at an individual level. Whilst we will be able to explore aggregate changes in these measures, it is possible that planned changes or random variations in intake of patients will limit our ability of the study to demonstrate the impact of peer-networks on these measures.

Nonetheless, we believe that this study provides a rare opportunity to examine the impact of a peer-led quality improvement initiative on the quality of inpatient mental health services. The findings will also add to our understanding of the impact of this widely used approach to improve the quality of health services.

### Status of the trial

Recruitment of the study commenced in June 2012 and ended in July 2014. Seventy-five wards were recruited in total and the data collection for the follow-up assessment was competed in October 2015. Data management and cleaning is currently ongoing and the results will be published by the end of 2016. The results will also be summarised and made available to the members of the participating units on the Royal College of Psychiatrists’ website.
